# Cell Intrinsic Deregulated ß-Catenin Signaling Promotes Expansion of Bone Marrow Derived Connective Tissue Type Mast Cells, Systemic Inflammation, and Colon Cancer

**DOI:** 10.3389/fimmu.2019.02777

**Published:** 2019-12-03

**Authors:** Abdulrahman Saadalla, Mariana Machado Lima, Funien Tsai, Abu Osman, Mahendra Pal Singh, David R. Linden, Kristen L. Dennis, S. M. Mansour Haeryfar, Michael F. Gurish, Fotini Gounari, Khashayarsha Khazaie

**Affiliations:** ^1^Department of Immunology, Mayo Clinic, Rochester, MN, United States; ^2^Robert H. Lurie Comprehensive Cancer Center, Feinberg School of Medicine, Northwestern University, Chicago, IL, United States; ^3^Department of Physiology and Biomedical Engineering, Mayo Clinic, Rochester, MN, United States; ^4^Department of Microbiology and Immunology, Schulich School of Medicine & Dentistry, Western University, London, ON, Canada; ^5^Division of Rheumatology, Immunology and Allergy, Brigham and Women's Hospital and Harvard Medical School, Boston, MA, United States; ^6^Section of Rheumatology, Department of Medicine, Knapp Center for Lupus Research, University of Chicago, Chicago, IL, United States

**Keywords:** mast cells, ß-catenin, bone marrow, proteases, inflammation, cancer

## Abstract

Mast cells constitutively express ß-catenin and expand in solid tumors such as colon and skin cancer. However, the role of ß-catenin signaling in mast cells and the cause or effect of mast cell expansion and tumor growth has yet to be established. In earlier studies we used mast cell depletion and protease staining approaches, to provide evidence for a causative role of mast cells in small bowel polyposis, and related specific phenotypes and distributions of tumor infiltrating mast cells to stages of tumor growth. Here we report that, stabilization of ß-catenin expands mast cells to promote high incidence of colon polyposis and infrequent small bowel polyps and skin cancer. Expression of a dominant acting ß-catenin in mast cells (5CreCAT) stimulated maturation and expression of granule stored proteases. Both mucosal and connective tissue type mast cells accumulated in colonic small bowel polyps independent of gender, and mice developed chronic systemic inflammation with splenomegaly. Reconstitution of polyposis-prone mice with bone marrow from 5CreCAT mice resulted in focal expansion of connective tissue like mast cells, which are normally rare in benign polyps and characteristically expand during adenoma-to-carcinoma transition. Our findings highlight a hitherto unknown contribution of ß-catenin signaling in mast cells to their maturation and to increased risk of colon cancer.

## Introduction

Two distinct populations of mast cells (MCs) have been described, the connective tissue mast cells (CTMCs) that are often embryonic-derived and serve as innate tissue sentinels, and the bone marrow-derived mucosal mast cells (MMCs) that are induced in response to parasitic invasion or tissue damage (1). CTMCs are independent of T-cells while MMCs are recruited into the gut as progenitors and mature in a T-cell dependent manner (2–4). In addition to their role in parasitic infections and allergic reactions, MC infiltration of tumors has been documented in mouse models of cancer and in human colon cancer (5, 6). However, the role of MCs in tumor growth is unclear (7). This could be related to MC plasticity and to the multitude of functions attributed to them. Indeed, there is evidence that MCs can both promote and reject tumors, and it has been suggested that MCs function as a rheostatic to regulate tumor immunity (8–10). Some striking examples of the protective function of MCs in cancer include a recent study where the genetic ablation of ACKR2, a decoy chemokine receptor, in the Min-APC mouse model of polyposis increased recruitment of MCs and led to tumor rejection in a MC and CD8 dependent manner (11). MCs were also reported to reject tumors in mouse models of metastatic colon cancer, in a TH9-dependent manner (12). Signals that activate MC maturation in the intestine independently of invading pathogens are poorly understood. Our findings demonstrated that initiation and progression of solid tumors are one such example. In earlier studies we used mouse models of spontaneous polyposis and intestinal cancer to identify distinct populations of tumor infiltrating MCs, which phenotypically resembled MMCs and CTMCs. MMCs expanded inside tumors during tumor initiation and CTMCs during tumor progression (5, 13). We also used MC depletion and MC protease knock-out experiments to demonstrate the role of MMCs in polyposis and of CTMCs in tumor growth, and additionally provided evidence for differential cytokine requirements of these two types of MCs (5, 13–15). What makes MCs reject tumors or promote tumor growth remain unknown.

MCs share many characteristics with stem cells including expression of the stem cell factor receptor c-Kit and ß-catenin. *In vitro* studies using cultured MCs demonstrated that activation of c-Kit feeds to the downstream canonical Wnt/ß-catenin signaling pathway (16, 17), and mediates MC maturation (18). Wnt proteins are abundantly expressed and secreted by colon tumor cells (19), and potentially could activate ß-catenin signaling in tumor infiltrating MCs. To better understand how ß-catenin signaling in MCs alters their properties, we expressed Catnb^lox(ex3)^ (20) a conditional dominant-stable ß-catenin in C57BL6/J mice. This was achieved by using Mcpt5-Cre (21), a MC specific Cre, to excise phosphorylation sites in exon-3 of the endogenous ß-catenin gene, thus preventing ubiquitination and degradation of ß-catenin. Nearly all the resulting Mcpt5-Cre Catnb^lox(ex3)^ mice (abbreviated as 5CreCAT) developed colonic polyps, independent of gender. Infrequent skin tumors and small bowel adenomatous polyps were also observed. Colonic polyposis coincided with notable intrapolyp expansion of both MMC and CTMC type MCs along with type-2 innate lymphoid cells (ILCs) and T-helper-2 T-cells in the colon and with systemic inflammation. Introduction of 5CreCAT bone marrow into irradiated, polyposis-prone APC^Δ468^ mice caused focal expansion of CTMC like MCs in the lesions and increased tumor load. These findings mechanistically link the action of ß-catenin in MCs to their gain of tumor promoting properties.

## Materials and Methods

### Primary Mast Cell Cultures

Femurs and tibia from 5Cre or 5CreCAT mice were flushed with PBS using 27-gauge needles and were dispersed by pipetting up and down. Cells were filtered through 40 μM strainers into 15 ml tubes. Cells were then centrifuged at 1,400 rpm for 10 min at 4°C, then resuspended and washed. Erythrocytes were lysed in ACK lysis buffer (Lonza) for 1 min. Lysis reaction was stopped with 2% BSA containing PBS and cells were then washed. Cells were resuspended in RPMI 1640 complete media (Lonza). RPMI 1640 media supplemented with 10% fetal bovine serum (Millipore), L-glutamine (2 mM), non-essential amino acids (0.1 mM), penicillin/streptomycin (100 U/ml), interleukin-3 (5-ng/ml), ß-Mercaptoethanol 7 ul, (Invitrogen), stem cell factor (12.5 ng/ml) (Gibco), and transferred into a 25-cm^2^ flask. On the next day, cells were split into three new 25-cm^2^ flasks filled with 5 ml of growth medium. A fresh 5 ml of medium was added every 48 h until the volume reached 15 ml. The cells were then centrifuged at 200 rcf and resuspended into 5 ml of growth medium, repeating the cycle. After 3 weeks of culture, cells were checked for maturity by microscopic morphology, cytospin staining for mast cell specific proteases, and by flow cytometry for the expression of ß-catenin, FcεR1, and c-Kit.

### β-Hexosaminidase Release (Mast Cell Degranulation) Assay

Mouse anti-DNP immunoglobulin E (IgE) at 1 μg/ml concentration was added overnight to primary bone marrow derived MC. On the next day, cells were washed (centrifugation at 1,000 rpm) with Tyrode's buffer, and subsequently challenged with DNP-BSA (Sigma) at 100 ng/ml for 30 min. The supernatant was collected and stored at 4°C and the pellet was lyzed with 0.1% Triton X. The 20 μl of supernatant or pellet lysate were incubated with 1 mM 4-nitrophenyl N-acetyl-β-d-glucosaminide (PNAG) for 60 min at 37°C and the reaction was stopped with 200 μl carbonate buffer (0.1 M, pH 10). β-hexosaminidase release in the supernatant was measured at 405 nm absorbance and interpreted as the percentage of total cellular (lysate + supernatant) β-hexosaminidase.

### Immunofluorescence and Immunohistochemistry

For tissue pathology assessment, both small bowel and large bowel were affixed in Swiss-roll fashion, fixed in 10% neutral-buffered formalin for overnight, and paraffin embedded and processed. For hematoxylin and eosin (H&E), immunofluorescence, and immunohistochemistry staining, 5 μm thick sections were dewaxed and then hydrated using xylene and alcohol/water. For immunofluorescence and immunohistochemistry, antigen retrieval was performed using Antigen Deloaker (Biocare medical) and Target Retrieval Solution (Dako). Following retrieval, tissues were washed with PBS and blocked using Background Sniper (Biocare medical), and Avidin/Biotin blocking kit (Vector Laboratories). Primary antibodies were prepared in Dako Antibody Diluent (Dako) and incubated overnight at 4°C. Slides were washed with Dako Wash buffer for 5 min 3 times. Secondary biotinylated antibodies (Vector laboratories) were then added and incubated for 45 min, and then Avidin-biotin complex with conjugated alkaline phosphate (Vector laboratories) was for 45 min. SIGMA*FAST*™ Fast Red TR/Naphthol (Sigma) was used for the color reaction. Slides were mounted with CC-mount (Sigma). GATA3 staining methods were adapted from Mackley et al. (22).

### Cytospin Tryptase Immunocytochemistry

MCs were cytospun on to slides at 500 rpm. The slides were dried for 5 min and then fixed with 2% paraformaldehyde for 10 min. Endogenous avidin and biotin were blocked using Avidin-biotin blocking kit (Vector Labs) and non-specific Fc-receptor binding was blocked by anti-mouse CD16/CD32 (clone 2.4G2). Staining steps were then completed as mention above.

### Antibodies

Mast cell protease antibodies (Rabbit anti-mouse mMCP-2, dilution: 1/200; Sheep anti-mMCP2, dilution: 1/200; Rabbit anti-mouse mMCP5, dilution: 1/100; Rabbit anti-mouse mMCP6, dilution: 1/500) were a gift from Dr. Michael Gurish. Antibodies to ß-catenin (clone 14, dilution: 1/200,) was purchased from BD Biosciences. CD3ε (clone eBio500A2, dilution: 1/100), and GATA3 (clone TWAJ, dilution: 1/50), were purchased from ebiosciences-Thermo. Alexafluor conjugated (anti-rat, anti-rabbit, anti-sheep, dilution: 1/200) were obtained from Invitrogen-Thermo. For flow cytometry antibodies, anti-CD11b (clone M1/70, dilution: 1/700), anti-CD11c (clone N418, dilution: 1/100), anti-Ly6C (clone HK1.4, dilution: 1/300), anti-Ly6G (clone 1A8, dilution: 1/300), anti-CD117 (clone 2B8, dilution: 1/200), and antiFcεR1 (clone MAR-1, dilution: 1/100) antibodies were purchased from Biolegend. Anti-RORγt (clone Q31-378, dilution: 1/100) was purchased from BD. Anti-Foxp3 (clone FJK-16s, dilution: 1/100) was purchased from ebiosciences-Thermo.

### Flow Cytometry

Cells were harvested from tissues of interest (spleens and MLN). 2 × 10^6^ cells per 100 μl staining volume were transferred to a 96-well, round-bottomed plate (Corning). After centrifugation for 5 min at 4°C at 200 rcf, cells were then incubated for 20 min on ice with Fc-block (clone 2.4G2) added and washed with PBS. Cells were then incubated with surface antibodies and LIVE/DEAD Fixable Blue stain (Molecular Probes) for 30 min in the dark on ice, and washed twice with PBS. Stained cells were then fixed using 2% PFA for 10 min in the dark on ice, and then washed once with PBS. For intracellular staining, cells were fixed and permeabilized using Foxp3/Transcription Factor Staining Buffer kit (eBioscience). Antibodies for intracellular staining were diluted in 100 μl 1X wash/perm buffer and cells were incubated in 100 μl of the staining solution for 2 h on ice or overnight at 4°C. Cells were then centrifuged and washed twice using the wash/perm buffer, and transferred to tubes for acquisition.

### Flow Cytometry Acquisition and Analysis

Cells were suspended at 2 × 10^6^ cells per 250-μl volume, and samples were run on a BD LSRII flow cytometer (BD Biosciences) and Accuri BD flow cytometer (BD Biosciences). Data were analyzed using FlowJo software (TreeStar).

### Microscopy

A Leica light microscope mounted with a Zeiss Axiocam 503 camera was used for imaging immunohistochemistry staining. For imaging fluorescent staining, a Zeiss Observer Z1 microscope mounted with an Axiocam 506 mono camera was used. Fiji software was used for image analysis.

### Bone Marrow Reconstitution Experiments

At 2 months of age, mice were irradiated with Cesium-137 (Cs) 1,100-rads dose (split over 2 days; 550 rads/day). Four hours following the irradiation, 2–4 × 10^6^ washed, RBCs lysed bone marrow cells obtained from control 5Cre or 5CreCAT mice were injected into the retro-orbital sinus. Mice were kept on antibiotics supplied in drinking water for 2 months.

### Statistical Analysis

All results are presented as the mean with SD. Significance between two groups was assessed by the Student's two-tailed *t*-test. *P*-value that was less than 0.05 was considered statistically significant for all data sets. All statistical analysis was performed with the use of Prism 8 software (GraphPad).

### Ethical Statement

Experiments with mice were approved by Institutional Animal Care and Use Committee protocol number A00004708-19 and A733113, Mayo Clinic.

## Results

**Figure 1:**
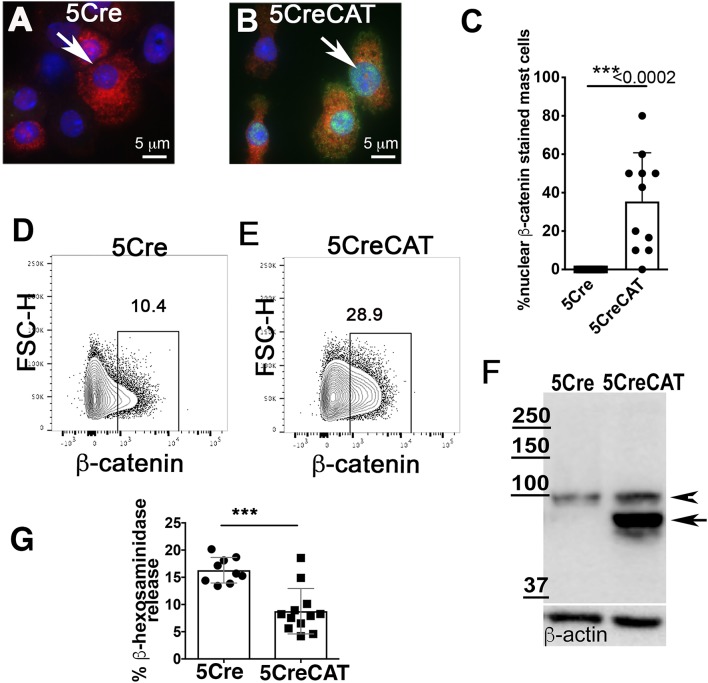
*Ex vivo* maturation of 5CreCAT MCs expressing a stabilized ß-catenin. **(A,B)** Bone marrow-derived MCs were cytospun and stained after 21 days of culture, for mMCP-6 (red) and nuclear ß-catenin (green); arrows point to nuclei, 630X. **(A)** IF stain of ß-catenin in negative control 5Cre MCs. **(B)** IF stain of ß-catenin in 5CreCAT MCs. **(C)** Frequency of IF stained MCs with detectable nuclear ß-catenin; unpaired Student's *t*-test, *p* < 0.0001. **(D,E)** Flow cytometry analysis of *ex vivo* matured 5Cre and 5CreCAT bone marrow-derived MCs, on day 21 of culture; the entire culture was gated for live cells and plotted as FSC-H vs. ß-catenin. **(F)** Western blot of extracts from 5Cre and 5CreCAT MCs. Note expression of a larger unmutated ß-catenin (arrow head) and a smaller exon-3 deleted and stabilized ß-catenin (arrow) in heterozygous 5CreCAT MCs. **(G)** ß-hexosaminidase degranulation assay of *ex vivo* matured 5Cre and 5CreCAT bone marrow derived MCs; unpaired Student's *t*-test, ^***^p < 0.0002.

To understand how ß-catenin shapes MC properties, we crossed Mcpt5-Cre mice (21) to Catnb^lox(ex3)^ mice, which express a conditional dominant ß-catenin (20). In the Mcpt5-Cre transgenic mice, the Cre recombinase is expressed specifically in MCs under the control of the promoter of mMCP5, a CTMC-specific protease (21, 23). We crossed the Mcpt5-Cre mice with Catnb^lox(ex3)^ mice to excise exon 3 of ß-catenin in MCs. Our earlier studies had shown that stabilization of ß-catenin has pro-inflammatory, cytotoxic, and oncogenic outcomes in T-cells. For example, CD4Cre Catnb^lox(ex3)^ mice that stabilized ß-catenin in T-cells had reduced numbers of mature T-cells and developed systemic inflammation and colitis, or T-cell leukemia (24, 25). To ascertain stabilization of ß-catenin in MCs, and their viability and functionality, we expanded MCs *ex vivo* from bone marrows of control Mcpt5-Cre mice (abbreviated to 5Cre) and Mcpt5-Cre Catnb^lox(ex3)^ mice (abbreviated to 5CreCAT) in medium containing SCF and IL-3. Matured MCs were identified by their morphology and by staining for mMCP6 containing storage granules. Immunofluorescence staining of cytospun cells detected co-expression mMCP6 and nuclear ß-catenin in less than half of the 5CreCAT cells, and positive mMCP6 staining but no ß-catenin in the control 5CreCat cultures (Figure 1A compare to Figure 1B, quantification in Figure 1C). FACS analysis detected expression of ß-catenin in both cultures, but a notable increase in 5CreCAT compared to 5Cre cells (Figure 1D compare to Figure 1E). To test for expression of the exon-3 deleted Catnb^lox(ex3)^ transgene (20), we performed Western blot analysis of cell extracts prepared from heterozygous 5CreCAT MCs, using antibody to ß-catenin. Control 5Cre MCs expressed a ~94 kD band corresponding to the native unmutated ß-catenin, while 5CreCAT MCs expressed an additional shorter prominent band corresponding to exon-3 deleted ß-catenin (Figure 1F). The matured 5CreCAT MCs expressed *bona fide* MC lineage markers c-Kit and FcεRI, and distributed into 3 putative stages of MC maturation described earlier (26) (Supplementary Figures 1A,B). Earlier reports have suggested that ß-catenin positively regulates the expression of granule stored proteases in MCs (18). Cross linking of cell surface IgE receptors triggered degranulation by both types of MC, but less efficiently by 5CreCAT than control 5Cre MCs. This could suggest either a delay in maturation or a partial loss of function of 5CreCAT relative to 5Cre MCs (Figure 1G).

**Figure 2:**
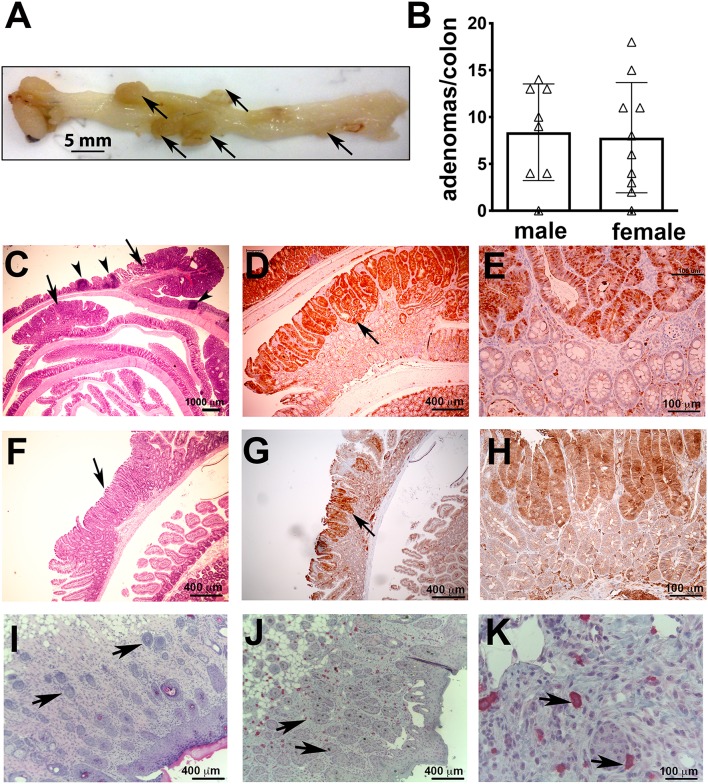
Colon polyps in 5CreCAT mice. **(A)** Representative colon of an aged 5CreCAT mouse; arrows point to individual polyps. **(B)** Number of colon polyps from 8 male and 10 female 5CreCAT mice. **(C–H)** Serially cut 5 μm sections of FFPE-fixed colons from 5.5 months old mice were used for histology and immunohistochemistry; arrows point to adenomatous polyps and arrow heads to lymphoid aggregates. Student's *t*-test did not show any significance. **(C)** Jelly roll of a representative 5CreCAT colon, H&E 16X. **(D)** Adenomatous polyp of the same, stained for ß-catenin, 50X. **(E)** Border of transformed and hyperplastic crypts in the same adenoma, stained for nuclear ß-catenin 200X. **(F)** Rare small bowel adenoma; H&E 50X. **(G)** the same adenoma, stained for ß-catenin of 50X. **(H)** Border of transformed and hyperplastic crypts in the same adenoma, stained for ß-catenin 200X. **(I)** 5CreCAT mouse skin tumor H&E stain 100X; arrows show nodules invading the dermis. **(J)** the same skin tumor mMCP5 stain 100X; arrows point to MCs. **(K)** the same image 400X.

Activated MCs can mediate colitis (27) or exacerbate ileum food anaphylaxis (28). We have reported extensively on focal intra-polyp and intra-tumor mastocytosis during polyposis and cancer in the small bowel and colon (5, 13–15). Therefore, we examined the 5CreCAT mice for intestinal pathologies. Younger mice appeared healthy, however both male and female mice developed rectal prolapse as early as 5 months of age. Necropsy of the mice determined that by 5.5 months of age, 9/10 male and 9/10 female 5CreCAT mice had large colonic polyps (Figures 2A,B), ranging in size between 1.5 and 7.0 mm with a mean diameter of 3.8 mm. Histological examination of FFPE sections of colon jelly rolls revealed pedunculated/tubular adenomatous polyps (Figure 2C). To confirm epithelial transformation, colon sections were stained with antibody to ß-catenin. Nuclear accumulation of ß-catenin was prominent in aberrant epithelial cells (Figures 2D,E). By contrast, small bowel adenomatous polyps were rare, and occurred in 1/10 of the examined mice. These lesions had a sessile/villous appearance typical of the small bowel adenomas in mouse models of hereditary polyposis (Figure 2F), and were otherwise similar to the colon adenomas with typical top-down morphology of aberrant epithelial cells (Figures 2G,H). MCs have also been associated with skin tumors (29). We detected only rare incidences of skin tumor, in 2/20 examined mice. These were squamous epithelial tumors that originated in the epidermis and produced nodules that extended deep into the dermis (Figure 2I). The nodules were infiltrated with mMCP5 granule-stored MCs (Figures 2J,K). This is consistent with earlier reported role of CTMCs in the initiation and progession of skin cancer (30). We conclude that chronic activation of ß-catenin in MCs imparts pathogenic properties to MCs, rendering them capable of initiating colon cancer with high prevalence, and increasing the risk of cancer in the small bowel and skin.

**Figure 3:**
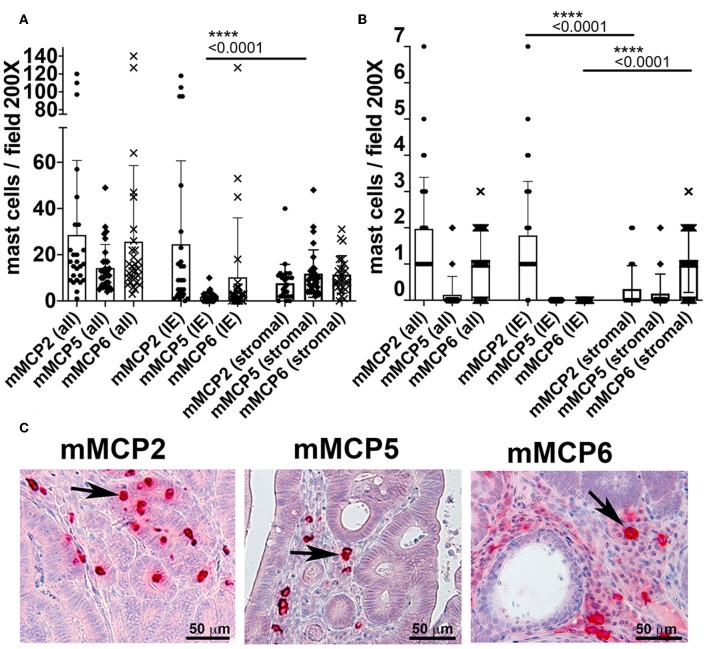
Quantitation of mast cells in colon polyps of 5CreCAT mice. **(A–C)** Distribution of MCs expressing mMCP2, 5 or 6 in colon adenomas of 5CreCAT mice; 200X field, *n* = 5 mice; plots show all mast cells (all), intraepithelial mast cells (IE), and stromal mast cells (stromal); unpaired Student's *t*-test. **(A)** Densities of MCs expressing mMCP2, 5, or 6 in spontaneous colon polyps; *n* = 5 mice. **(B)** Densities of MCs in distant healthy colon; *n* = 4 mice. **(C)** Representative MC stains in serial sections of 5CreCAT colon-adenomas, for mMCP2, 5, or 6.

To better define the status of MCs in 5CreCAT mice, we stained histological sections of the colon with antibodies to MC-specific proteases. In the healthy gut, storage granule content of mMCP2 and intraepithelial localization identifies MMCs while mMCP5 and mMCP6 together with stromal localization identifies CTMCs (2–4). While we cannot rule out that tumor infiltrating MCs may be different from MCs that infiltrate the healthy gut, it was intriguing to detect in 5CreCAT colon adenomas, MCs with both MMC and CTMC characteristics. It was also of significance that MC densities in these lesions were at least 10 times higher than in the healthy adjacent, or marginal tissue (Figure 3A compare to Figure 3B). mMCP5 was strongly expressed in stromal MCs, while mMCP2 and mMCP6 were expressed by both intraepithelial and stromal MCs (Figure 3C), as has been described for the healthy gut responding to nematode infection. In earlier studies, we established that adenomatous polyps in mouse models of polyposis are from onset infiltrated by mMCP2^+^ intraepithelial MMCs while transition of adenomas to carcinomas is marked by accumulation of stromal mMCP6+ and submucosal mMCP5+ CTMCs (13). Therefore, the high densities of mMCP5^+^ and mMCP6^+^ MCs in 5CreCAT mice had higher densities of both stromal and intraepithelial mMCP5^+^ MCs than age matched TS4Cre APC^lox468^ (cAPC) mice that develop polyposis due to loss of function of APC in gut epithelial cells (Supplementary Figure 2). The high densities of mMCP5^+^ MCs in 5CreCAT polyps was surprising and distinguished these from typical benign polyps that arise spontaneously in mouse models of polyposis.

**Figure 4:**
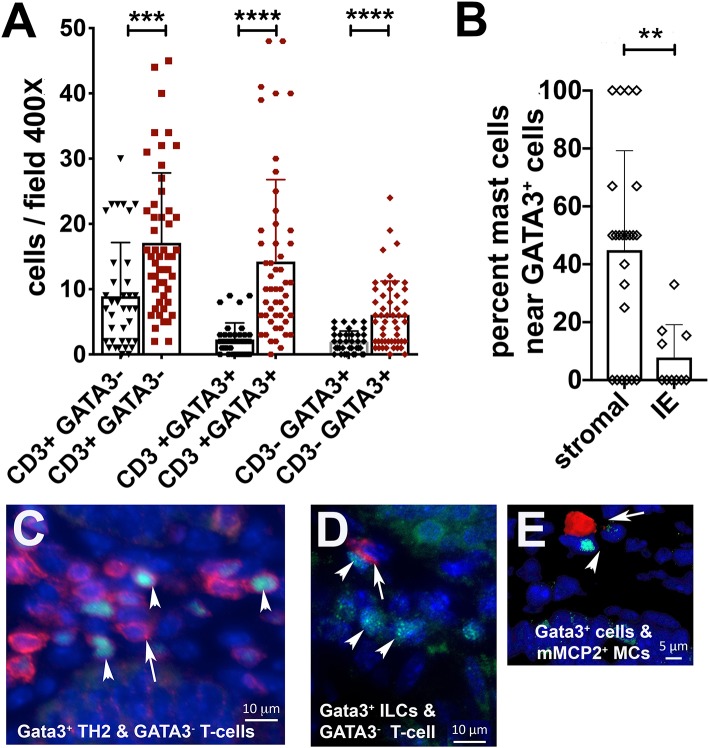
Co-expansion of mast cells, type-2 innate lymphoid cells, and TH2 cells in 5CreCAT colons. **(A)** Densities of T-cells (CD3^+^GATA3^−)^, TH2 cells (CD3^+^GATA3^+^), and ILC2s (CD3^−^GATA3^+^) infiltrating colon adenomas (brown) and adjacent healthy colon (black), in 5CreCAT mice; 5 μm section, 400X field, *n* = 3 mice per group. **(B)** Percent of total adenoma infiltrating MCs within one cell diameter distance of GATA3^+^ cells; average of 5 adenomas. **(C)** Representative images of CD3 (red), GATA3 (green), and DAPI (blue) stained cells in a colon adenoma; 630X. Arrows point to CD3 stained cells, arrowheads point to GATA3 stained cells. **(D,E)** Representative image of mMCP2 expressing MCs (red), GATA3 stained cells (green), and DAPI stained cells (blue) in colon adenoma; 630X. Arrow points to mMCP2, arrow head to GATA3. Significance in **(A–C)** was calculated by unpaired Student's *t*-test, ^****^p < 0.0001, ^***^p < 0.001, ^**^p < 0.0024.

We had also earlier reported that spontaneous polyposis in genetically susceptible APC deficient mice is marked by mixed TH2 and TH17 inflammation, and that MCs and GATA3 positive cells tend to accumulate together inside polyps (13). To further investigate TH2 inflammation in 5CreCAT polyps we performed IF staining on FFPE sections for the Gata3^+^CD3^−^ innate lymphoid type-2 cells (ILC2s), Gata3^+^CD3^+^ T-helper cells type-2 cells (TH2) and, CD3^+^ GATA3^−^ T-cells. We detected preferential intra-polyp accumulation of all three cell types, over and above the densities of the same cells in distant healthy tissues (Figures 4A,C,D). Thus, the 5CreCAT adenomas had a strong TH2 microenvironment supportive of MC expansion. Interestingly, nearly half of the polyp infiltrating mMCP2^+^ MCs were located within one cell distance of GATA3^+^ cells (Figures 4B,E). This finding raises the intriguing possibility of close interactions of MCs and ILCs within the polyps. This finding raises the intriguing possibility of close interactions of MCs and ILCs within the polyps.

**Figure 5:**
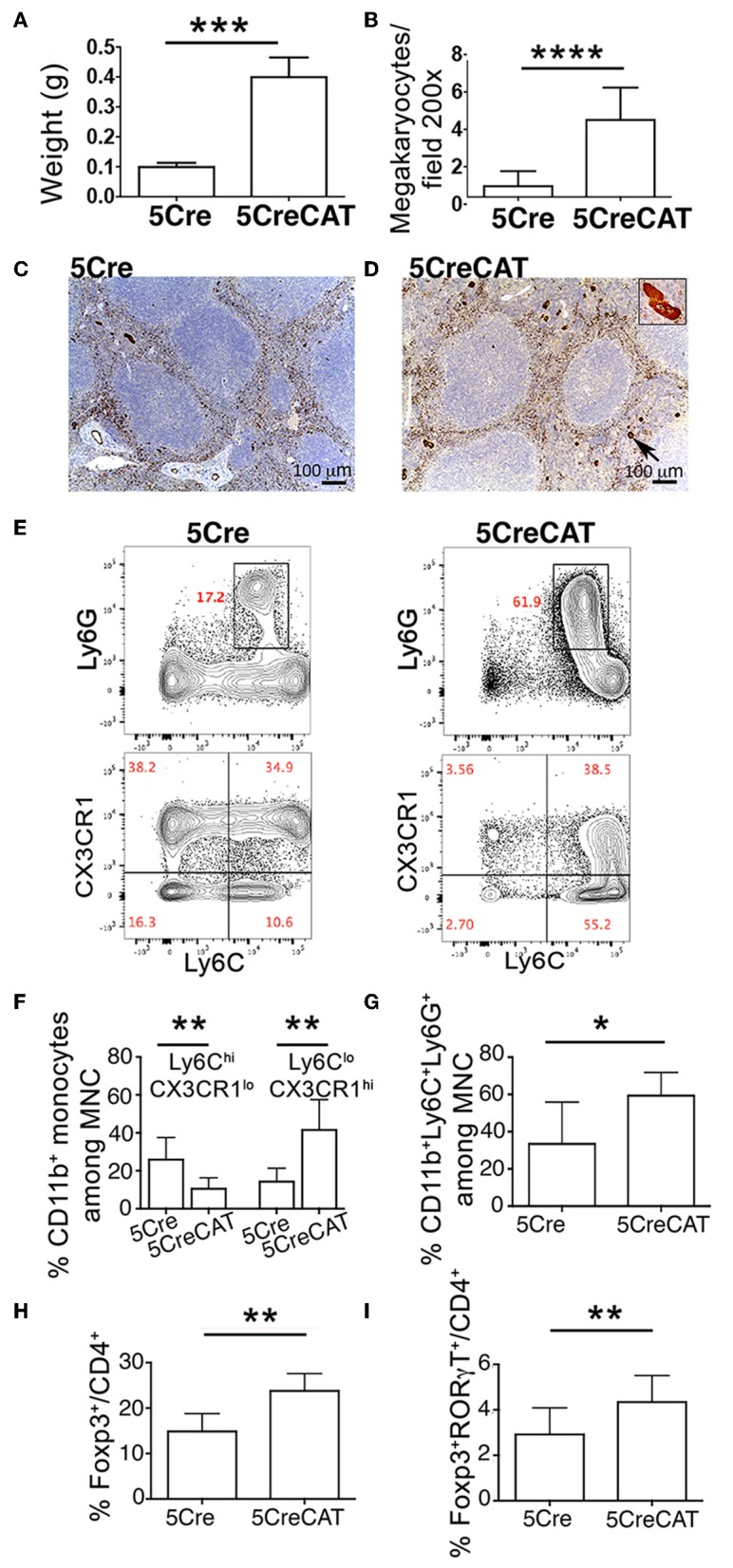
Systemic inflammation in 5CreCAT mice. **(A)** Average spleen weight of 5Cre and 5CreCAT mice at 5.5 months of age. **(B)** Density of megakaryocytes in spleens of healthy and 5CreCAT mice, measured by vWF staining of tissue sections. **(C,D)** vWF-stained megakaryocytes in spleens of control 5Cre and 5CreCAT mice respectively; 100X. Insert in “D” is an enlarged image of a vWF stained megakaryocyte. **(E)** Gating of CD11b^+^ splenocytes for CX3CR1^lo^ Ly6C^hi^ inflammatory and CX3CR1^hi^ Ly6C^lo^ resident monocytes. **(F)** Densities of spleen infiltrating CD11b^+^ CX3CR1^lo^ Ly6C^hi^ inflammatory and CD11b^+^ CX3CR1^hi^ Ly6C^lo^ resident monocytes, measured by flow cytometry. **(G)** Frequencies of Ly6C^+^Ly6G^+^ myeloid derived suppressor cells in the spleen. **(H)** Treg to effector CD4^+^ T-cell ratio in the mesenteric lymph nodes of control 5Cre and of 5CreCAT mice. **(I)** RORγt^+^ Tregs relative to effector CD4^+^ T-cell in mesenteric lymph nodes, measured by flow cytometry. ^*^p < 0.1; ^**^p < 0.01; ^***^p < 0.001; ^****^p < 0.0001, unpaired Student's *t*-test. Age matched 5.5 months old 5Cre (*n* = 5) and 5CreCAT (*n* = 7) mice.

With age, both male and female 5CreCAT mice developed splenomegaly (Figure 5A). Spleens of 5CreCAT mice contained high densities of megakaryocytes as compared to control 5Cre mice (Figures 5B–D). Megakaryocytes are now recognized as immune cells capable of inducing inflammation (31). The 5CreCAT spleens also contained increased densities of pro-inflammatory Ly6C^hi^CX3CR1^lo^ monocytes, but decreased resident anti-inflammatory Ly6C^lo^CX3CR1^hi^ monocytes (32) (Figures 5E,F), and high densities CD11b^+^Ly6C^+^Ly6G^+^ myeloid-derived suppressor cells (Figure 5G). Regulatory T-cells respond to inflammation. We had reported earlier that during polyposis mast cell render Tregs pro-inflammatory and capable of secreting IL-17 (33). Accordingly, total densities of Tregs and the fraction of Tregs that co-expressed Foxp3 and RORγt were elevated in the MLN of 5CreCAT mice (Figures 5H,I).

**Figure 6:**
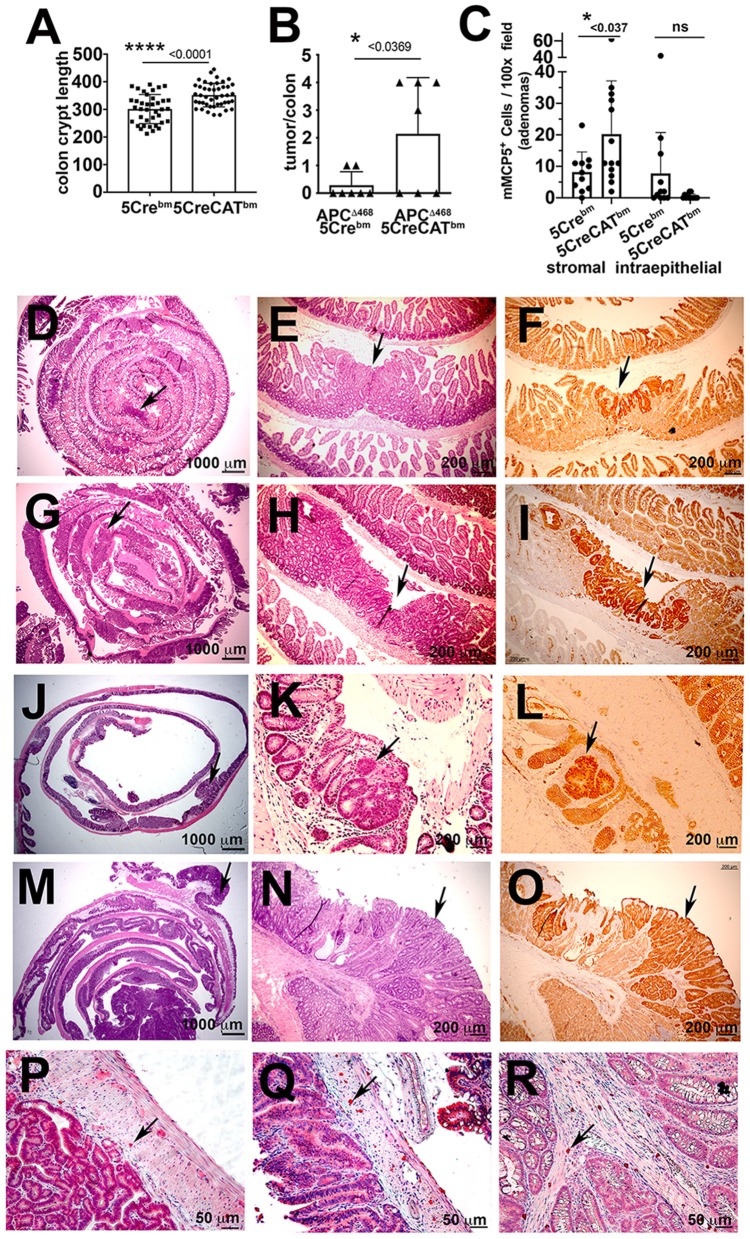
Transplant of 5CreCAT bone marrow in APC^Δ468^ mice worsens colon polyposis and promotes expansion of CTMC like stromal mMCP5^+^ MCs. Mice were bone marrow reconstituted at 8 weeks of age and aged to 5.5 months, at which time they were analyzed. **(A)** Colon crypt lengths in C57BL/6 mice reconstituted with control 5Cre or 5CreCAT bone marrow. Measurements were from H&E stained sections at 200X magnification, using the line tool in ImageJ; each mark represents one crypt **(B)** Numbers of colon polyps in APC^Δ468^ mice reconstituted with control 5Cre or 5CreCAT bone marrow; each mark represents one colon. **(C)** Densities of stromal or intraepithelial mMCP5 expressing MCs in the same polyps, counted in 100x fields; *n* = 4 mice per group. **(D–O)** Histology and immunohistology of 5 μm FFPE sections of adenomas. APC^Δ468^ mice were lethally irradiated at 8 weeks of age and reconstituted with bone marrow from 5Cre or 5CreCAT mice, aged to 5.5 months and then analyzed; arrows point to adenomas. **(D)** Representative jelly roll of proximal small bowel of control 5Cre bone marrow chimeric APC^Δ468^ mouse; H&E 16X. **(E)** Small bowel adenomatous polyp of the same; H&E 100X. **(F)** The same lesion; β-catenin stain 100X. **(G)** Representative jelly roll of proximal small bowel of 5CreCAT bone marrow chimeric APC^Δ468^ mouse; H&E 16X. **(H)** Small bowel adenomatous polyp of the same; H&E 100X. **(I)** The same lesion, β-catenin stain 100X. **(J)** Representative colon jelly roll of control 5Cre bone marrow chimeric APC^Δ468^ mouse; H&E 16X. **(K)** Colon adenomatous polyp of the same; H&E 100X. **(L)** The same lesion, β-catenin stain 100X. **(M)** Representative colon jelly roll of 5CreCAT bone marrow chimeric APC^Δ468^ mouse; H&E 16X. **(N)** Colon adenomatous polyp of the same; H&E 100X. **(O)** The same lesion, β-catenin stain 100X. **(P–R)** Representative mMCP5 stained adenoma infiltrating mast cells. **(P)** Mast cells in the submucosal of 5Cre chimeric APC^Δ468^ mouse colon adenoma. **(Q,R)** Mast cells in the submucosal and mucosal stroma respectively of 5CreCAT chimeric APC^Δ468^ mouse colon adenoma. Significance in A-C was calculated by unpaired Student's *t*-test.

To test the direct effect of 5CreCAT MCs on colon cancer, we introduced 5CreCAT or control 5Cre bone marrow into lethally-irradiated WT mice and polyposis-prone APC^Δ468^ mice 8 weeks after birth. We then aged the mice to 5.5 months of age. Mice that were reconstituted with 5CreCAT bone marrow had markedly elongated colon crypts as compared to mice that received 5Cre bone marrow (Figure 6A), demonstrating enhanced colonocyte renewal. APC^Δ468^ mice normally develop severe small bowel polyposis, but few if any colonic polyps. Accordingly, by 5.5 months of age, only 2/7 control 5Cre-chimeric APC^Δ468^ mice developed one colonic polyp each (Figure 6B). By contrast 4/7 5CreCAT-chimeric APC^Δ468^ mice developed 3–4 colonic polyps (Figure 6B). Histologic analysis revealed aggressive adenomas in 5CreCAT chimeric relative to 5Cre mice, with extensive nuclear ß-catenin staining throughout the lesions that extended to the submucosal border (Figures 6D–O). In earlier studies we had related expansion of CTMC to adenoma to carcinoma transition in mice (13). Accordingly, we noted expansion of stromal mMCP5 stained MCs in the 5CreCAT relative to 5Cre chimeric APC^Δ468^ mice (Figures 6C,P–R). These findings are consistent with tumorigenic properties of 5CreCAT bone marrow derived MCs.

## Discussion

We demonstrated that MCs tolerate over expression of ß-catenin which however predisposes mice to develop colonic polyps in an age dependent manner, independently of gender. Accumulation of both MMC and CTMC type MCs in the polyps distinguished these from benign adenomas that develop in mouse models of spontaneous polyposis, which are primarily infiltrated by mMCP2^+^ MMCs but not mMCP5^+^ CTMCs (5, 13). The frequent incidence of colonic polyps, but only rare small polyps, indicates a association between stabilization ß-catenin in MCs and increased risk of colon cancer. We provided further evidence in support of the tumorigenic properties of ß-catenin overexpressing MCs by generating bone marrow chimeric mice. APC^Δ468^ mice that received 5CreCAT bone marrow developed more aggressive colon polyposis. In both models, MC expansion was largely confined to tumors and was markedly less in the distant healthy tissue, suggesting a positive feedback loop between the tumor and MCs. Altogether, these observations strongly suggest that Mcpt5-Cre mediated stabilization of ß-catenin in 5CreCAT mice increases the risk of colon cancer by activating MCs.

A number of observations distinguish the 5CreCAT mice from classical mouse models of polyposis. Mouse models of hereditary polyposis including the APC^Δ468^ mice mainly develop small bowel polyps, and only rarely have polyps in the colon. In earlier studies we made the surprising observation that polyposis in the small bowel and colon has differential requirements for cytokines and MC. In small polyposis MMCs expanded in an IL10 dependent manner and both IL10 and MMCs were essential for polyp growth (5, 13, 15). By contrast, colon polyps were not affected by IL10 deficiency and progressed in IL10 deficient mice with extensive infiltration of the polyps with CTMCs (14, 15). The and intrapolyp expansion of mMCP5+ CTMCs in 5CreCAT mice is in agreement with these earlier observations, and with the notion that mMCP5+ CTMCs may have a causative role in colon carcinogenesis.

Colonic polyposis in 5CreCAT mice coincided with systemic inflammation, which we readily detected in the spleens of affected mice. The inflammation was characterized by splenomegaly, notable expansion of megakaryocytes, increase in frequencies inflammatory monocytes and myeloid derived suppressor cells, and expansion of Tregs. We had reported earlier that Treg suppressive properties are compromised in polyposis and colon cancer, as they gain TH17 like characteristics and become pro-inflammatory (25, 34–36). This change in properties coincided with upregulation of both the co-expression of Foxp3 and the canonical TH17 transcription factor RORγt (33, 34). Similar changes in Treg characteristics were introduced by *ex vivo* co-culture of Tregs with MCs (33). Accordingly, in the current study we detected increased densities of CD4^+^Foxp3^+^ and CD4^+^Foxp3^+^RORγt^+^ Tregs in the mesenteric lymph nodes (MLNs) of 5CreCAT mice. These findings indicate that expansion of MCs in the colon promotes systemic inflammation, and this could be in part by altering Treg properties.

Our finding that constitutive ß-catenin activity expands MCs is consistent with earlier *ex vivo* studies that documented Wnt5A-stimulation of MC maturation (18), a report suggesting that ß-catenin is activated downstream of c-Kit (16), and observations suggesting that ß-catenin signaling in MCs is inhibited through pharmacologic blockade of c-Kit phosphorylation (37). These reports together with our findings encourage more work in understanding the cross talk between c-Kit and ß-catenin signaling in MCs in colon cancer.

Activation of MCs by ß-catenin coincided with the expansion of Gata3^+^ type-2 lymphocytes. MCs are essential for induction of ILC2 responses to helminth infection (38). MC-ILC cooperation has been documented in animal models of skin scratch and food allergy ileitis (28), and expansion of MCs in the duodenum was reported to be ILC-dependent (28). While a TH2 environment in colonic polyps is consistent with MC expansion, the nature of communication between MCs and Gata3^+^ lymphocytes, which include T-cells and ILC2s is poorly understood. Activation of ß-catenin in MCs may define the nature and outcomes of such interactions. Further research is needed to establish whether the close apposition of Gata3^+^ cells and MCs in 5CreCAT polyps indicates paracrine crosstalk and/or direct contact between the cell types.

Tissue resident CTMCs are abundant in the skin, which expand in response to various stimuli including scratching (28), and have also been associated with skin tumors (29). While we expected skin tumors in 5CreCAT mice the relatively low incidence of skin tumors was a surprise. One possibility is that skin-residing CTMCs which are typically innate and of embryonic origin are distinct from the bone marrow derived ß-catenin activated-MCs that also happen to have a CTMC like phenotype.

The transfer of bone marrow from 5rCreCAT mice to APC^Δ468^ mice was sufficient to increase colonic polyposis and enhance their aggressive characteristics marked by extensive nuclear ß-catenin staining of the lesions. The transfer of 5CreCAT bone marrow to the APC^Δ468^ mice resulted in focal expansion of CTMCs in the tumor stroma, as compared to 5Cre bone marrow chimeric mice. These findings support the bone marrow origin of tumor promoting MCs in the 5CreCAT chimeric mice. Our efficiency of bone marrow reconstitution (assessed by reconstituting CD45.1 mice with CD45.2 bone marrow and analyzing MNCs from mesenteric lymph nodes) is typically over 85%. Tissue resident gut MC could be radio resistant. However, we have previously shown that with polyp growth, additional bone marrow derived MCp are actively recruited to the gut. By transferring CD34 CD43 deficient bone marrow to lethally irradiated APC^Δ468^ mice we were able to efficiently block MCp homing of bone marrow derived MCp to the gut, and attenuated polyposis (5). We are therefore confident that bone marrow derived mast cells contribute to polyposis. In the current study, bone marrow transfer from 5CreCAT mice to APC^Δ468^ mice increased colonic polyposis and the density of mMCP5^+^ mast cells in the polyp stroma. These findings indicate that expression of a dominant active ß-catenin conveys colon cancer promoting properties to bone marrow derived mast cells.

Overall, these observations provide new insights into the role Wnt/ß-catenin signaling in the gain of pro-inflammatory and colon cancer promoting properties by MCs. Our findings expand and solidify the role of mast cells in tumor immunology. Nevertheless, we are aware that in spite of similarities in expression of mMCP5, submucosal distribution, and association with tumor invasion, our observations do not prove that 5CreCAT MCs are identical to mMCP5^+^ CTMCs in healthy mice or in tumors. This question remains to be addressed in future studies by further molecular and functional comparisons of the MCs.

## Data Availability Statement

All datasets generated for this study are included in the article/Supplementary Material.

## Ethics Statement

The animal study was reviewed and approved by Mayo Clinic.

## Author Contributions

AS, ML, FT, AO, and MS participated in the design and execution of experiments, collected and analyzed data, helped in preparation of figures, and text of manuscript. DL, KD, SMMH, and FG provided material and critical advice, participated in the interpretation of findings and edited draft of manuscript. KK conceived the project, designed experiments, analyzed and interpreted data, wrote the manuscript. MG generously provided material and crucial advice without which this work would have not been possible.

### Conflict of Interest

The authors declare that the research was conducted in the absence of any commercial or financial relationships that could be construed as a potential conflict of interest.

## Supplementary Material

Supplementary Figure 1 Flow cytometry analysis of *ex vivo* matured 5Cre and 5CreCAT MCs. MCs were harvested on day 21 of culture and stained for ckit and FcεR1. FACS analysis shows mature MC (MC), early mature MC (eMC), and MC progenitors (MCp) as described by Bankova et al. (26).

Supplementary Figure 2 Quantitation of mMCP5 expressing MCs in colon adenomas of 5CreCAT mice relative to TS4Cre APClox468 (cAPC) mice at 5.5 months of age; *n* = 3 mice; unpaired Students *t*-test.

## Abbreviations

5Cre: Mcpt5-Cre mice
5CreCAT: Mcpt5-Cre Catnb^lox(ex3)^ compound mutant mice
MC: mature mast cells
eMCP: early mature mast cells
MCp: mast cell progenitors
rcf: relative centrifugal force
RBC: red blood cells
FFPE: formalin fixed paraffin embedded
MNC: mononuclear cells
CTMC: connective tissue mast cells
MMC: mucosal mast cells
IF: immunofluorescence:
PCR: polymerase chain reaction
ILC: innate lymphoid cells
TH2: T-helper cells type 2
MLNs: mesenteric lymph nodes
APC: adenomatous polyposis coli.
