# Supplementary figures and images for: Cell Intrinsic Deregulated ß-Catenin Signaling Promotes Expansion of Bone Marrow Derived Connective Tissue Type Mast Cells, Systemic Inflammation, and Colon Cancer
Source: Front Immunol. 2019 Dec 3;10:2777. doi: 10.3389/fimmu.2019.02777 (PMC6902090; doi:10.3389/fimmu.2019.02777)

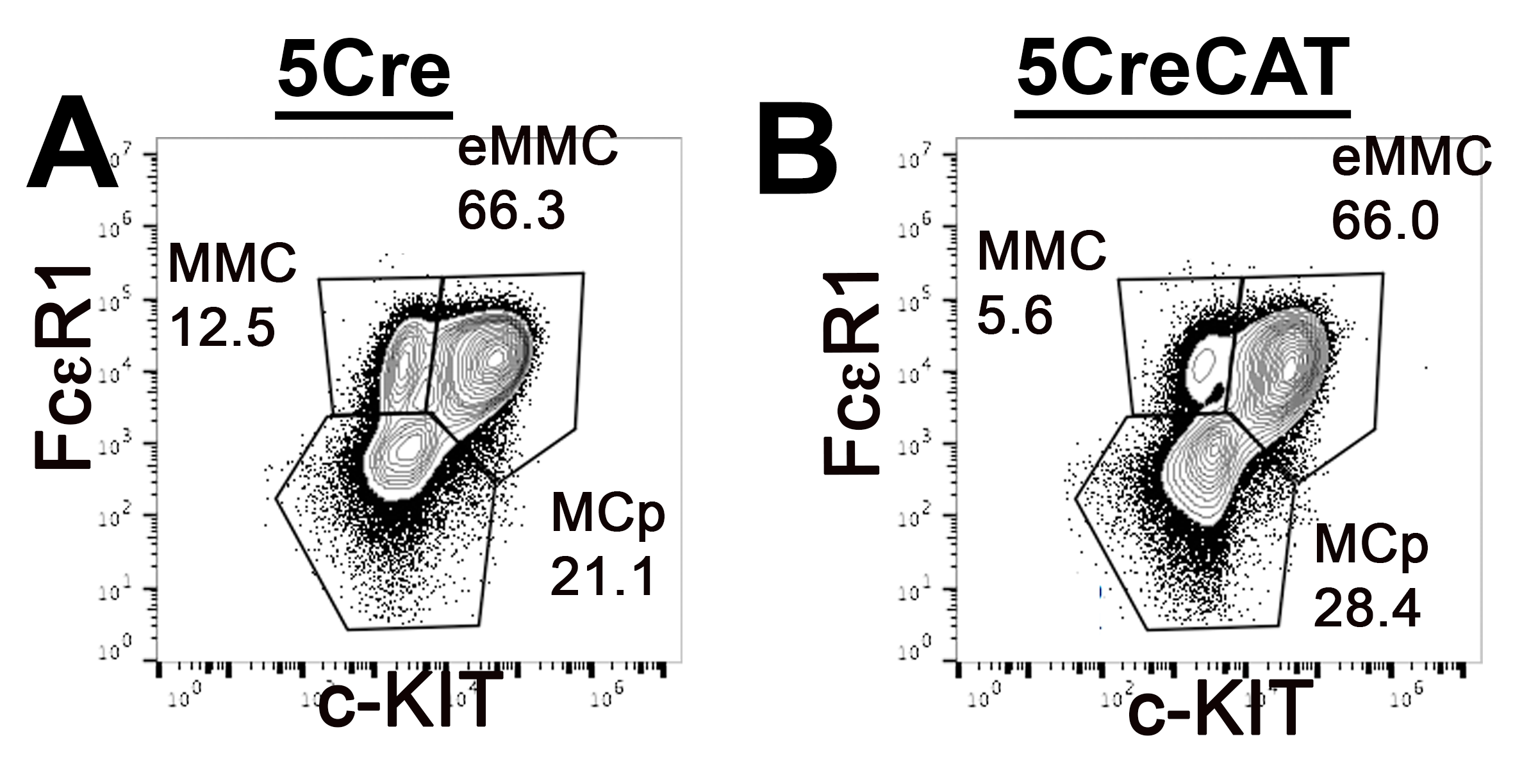

Supplement: Supplementary Figure 1 — Flow cytometry analysis of ex vivo matured 5Cre and 5CreCAT MCs. MCs were harvested on day 21 of culture and stained for ckit and FcεR1. FACS analysis shows mature MC (MC), early mature MC (eMC), and MC progenitors (MCp) as described by Bankova et al. (26). [file Image_1.TIF]

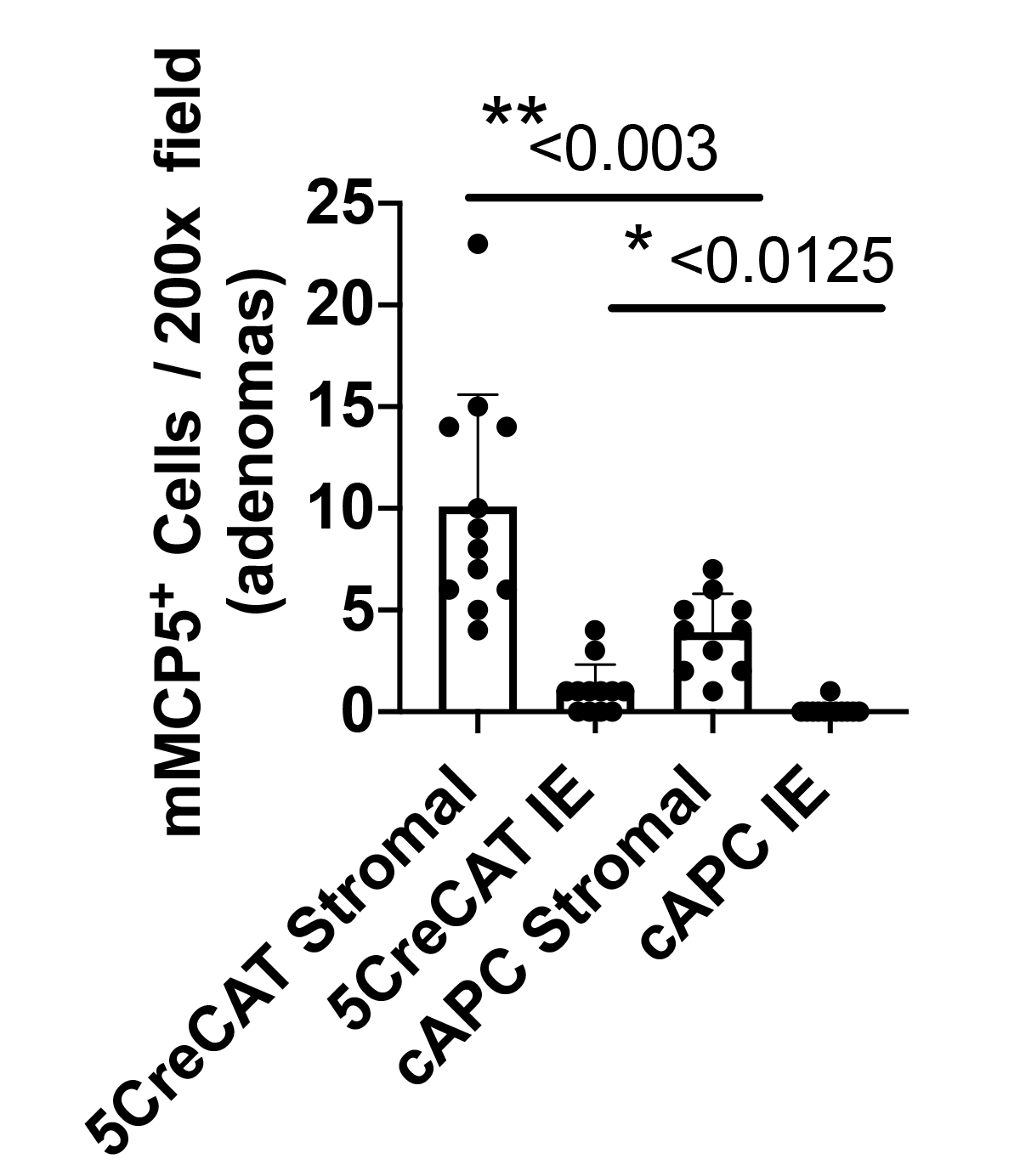

Supplement: Supplementary Figure 2 — Quantitation of mMCP5 expressing MCs in colon adenomas of 5CreCAT mice relative to TS4Cre APClox468 (cAPC) mice at 5.5 months of age; n = 3 mice; unpaired Students t-test. [file Image_2.TIF]
